# Integrated Multi-Omics Analysis Reveals the Survival Strategy of Dongxiang Wild Rice (DXWR, *Oryza rufipogon* Griff.) Under Low-Temperature and Anaerobic Stress

**DOI:** 10.3390/plants14203120

**Published:** 2025-10-10

**Authors:** Jilin Wang, Cheng Huang, Hongping Chen, Lijuan Tang, Dianwen Wang

**Affiliations:** Rice National Engineering Research Center (Nanchang), Rice Research Institute, Jiangxi Academy of Agricultural Sciences, Nanchang 330200, China

**Keywords:** Dongxiang wild rice (DXWR), seed germination, stress resistance, genome, transcriptome, metabolome

## Abstract

Dongxiang wild rice (DXWR, *Oryza rufipogon* Griff.), the northernmost known wild rice species, exhibits exceptional tolerance to combined low-temperature and anaerobic stress during seed germination, providing a unique model for understanding plant adaptation to complex environmental constraints. Here, we employed an integrated multi-omics approach combining genomic, transcriptomic, and metabolomic analyses to unravel the synergistic regulatory mechanisms underlying this tolerance. Genomic comparative analysis categorized DXWR genes into three evolutionary groups: 18,480 core genes, 15,880 accessory genes, and 6822 unique genes. Transcriptomic profiling identified 10,593 differentially expressed genes (DEGs) relative to the control, with combined stress triggering the most profound changes, specifically inducing the upregulation of 5573 genes and downregulation of 5809 genes. Functional characterization revealed that core genes, including *DREB* transcription factors, coordinate energy metabolism and antioxidant pathways; accessory genes, such as glycoside hydrolase *GH18* family members, optimize energy supply via adaptive evolution; and unique genes, including specific UDP-glycosyltransferases (*UDPGTs*), confer specialized stress resilience. Widely targeted metabolomics identified 889 differentially accumulated metabolites (DAMs), highlighting significant accumulations of oligosaccharides (e.g., raffinose) to support glycolytic energy production and a marked increase in flavonoids (153 compounds identified, e.g., procyanidins) enhancing antioxidant defense. Hormonal signals, including jasmonic acid and auxin, were reconfigured to balance growth and defense responses. We propose a multi-level regulatory network based on a “core-unique-adaptive” genetic framework, centered on *ERF* family transcriptional hubs and coordinated through a metabolic adaptation strategy of “energy optimization, redox homeostasis, and growth inhibition relief”. These findings offer innovative strategies for improving rice stress tolerance, particularly for enhancing germination of direct-seeded rice under early spring low-temperature and anaerobic conditions, by utilizing key genes such as *GH18s* and *UDPGTs*, thereby providing crucial theoretical and technological support for addressing food security challenges under climate change.

## 1. Introduction

Rice (*Oryza sativa* L.), a staple food for half the global population, faces significant challenges in direct seeding systems due to adverse environmental conditions during germination. Anaerobic and low-temperature stresses commonly cause impaired or inhibited germination, followed by uneven seedling emergence, and ultimately lead to yield losses [1,2,3,4]. Developing varieties with enhanced tolerance to these combined stresses is therefore critical for optimizing direct-seeded rice production.

Wild rice species (*Oryza rufipogon* Griff. and related taxa), as the ancestors of cultivated Asian rice, have evolved under diverse ecological pressures, resulting in rich genetic diversity and a wide range of stress resistance mechanisms that remain largely untapped in modern breeding programs [5,6]. These wild relatives thus serve as invaluable reservoirs of alleles for enhancing abiotic stress tolerance in cultivated rice. Among them, Dongxiang wild rice (DXWR, *Oryza rufipogon* Griff.) is particularly noteworthy due to its extreme northern distribution (28°14′ N) and documented resilience to multiple environmental constraints [6].

Under anaerobic conditions, rice employs coleoptile elongation as an escape strategy. This escape strategy is supported by transcriptomic reprogramming, which reveals a significant upregulation of genes involved in sucrose/starch mobilization (to provide energy and carbon skeletons), glycolysis and fermentation (to maintain energy production under hypoxia), cell expansion (to drive coleoptile elongation), and ethylene signaling (a key hormone promoting the escape response), while downregulating oxygen-dependent pathways such as aerobic respiration [7,8]. Key genetic determinants include *OsTPP7*, which promotes trehalose-6-phosphate turnover to fuel starch mobilization and coleoptile growth [9]. Hormonal crosstalk, particularly the antagonism between gibberellins (GAs) and abscisic acid (ABA), is central to regulating germination and dormancy under hypoxia [10,11]. A 14-3-3 protein from weedy rice interacts with OsHOX3 and OsVP1 to modulate ABA signaling, dramatically improving emergence under flooding [12]. Glucosyltransferase OsUGT75A further promotes tolerance by glycosylating ABA and jasmonic acid (JA), reducing free hormone levels and facilitating coleoptile elongation via OsJAZ-OsABI interactions [13]. Accumulation of specific amino acids also represents a conserved response to hypoxia [14,15].

Rice germination is highly sensitive to cold stress (<17 °C), leading to reduced germinability, delayed sprouting, and seedling mortality [1]. Low-temperature germination (LTG) is a quantitative trait. The cloned QTL *qLTG3-1* facilitates vacuole formation in seed coat and coleoptile epidermal cells, relaxing these structures to promote germination under cold [2,16,17]. Transcriptomics indicates LTG involves differential expression of genes enriched in starch metabolism and ABA response pathways, alongside hormone signaling, antioxidant defense, and carbohydrate metabolism [18]. For instance, elevated *OsUBC12* promotes degradation of ABA signaling regulator OsSnRK1.1, enhancing LTG in japonica rice [19]. OsNAL11 and OsBURP12 likely modulate ABA and cell wall modification [20], while OsMYB30 upregulates *OsTPP1*, leading to excessive trehalose accumulation that inhibits α-amylase (*OsAMY1a*) and suppresses germination [21]. Metabolomics further highlights divergent accumulations of sugars, amino acids, fatty acids, and flavonoids during cold stress [22,23], underscoring the interplay of genetic, metabolic, and hormonal regulation.

The long-term adaptation of DXWR to subtropical monsoon swamps suggests the evolution of unique tolerance mechanisms to the combined stress of low temperature and hypoxia [24,25,26,27]. Seed germination, being the most environmentally sensitive stage, determines emergence success in direct-seeded systems facing these dual stresses [28,29]. However, while studies have explored DXWR’s genetic diversity or responses to single stresses [30,31], its molecular regulatory network and metabolic remodeling mechanisms for coordinated adaptation to combined low temperature and hypoxia remain completely unknown. This knowledge gap severely hinders the exploitation of DXWR’s immense genetic potential for breeding stress-tolerant direct-seeded rice.

Multi-omics integration offers a powerful approach to understanding the plant’s complex adaptive strategies under a multi-stress environment. Comparative genomics reveals species-specific genetic variations [32], transcriptomics captures dynamic gene expression [33], and metabolomics identifies key physiological effectors [34]. Although single-omics studies exist for rice germination stress tolerance [30,35], a systematic, multi-dimensional analysis of wild rice adaptation, particularly under combined low-temperature and hypoxia, is lacking. The coordination of critical processes like energy metabolism reprogramming, reactive oxygen species (ROS) balance, and hormone signaling crosstalk under dual stress remains an unresolved scientific question.

This study utilizes DXWR to address this critical gap. We construct a simulated “low-temperature and anaerobic germination” system and employ an integrated comparative genomics, transcriptomics, and metabolomics approach to elucidate: (1) Unique genomic features (e.g., gene family expansions/functional differentiation) underlying DXWR’s stress adaptation; (2) How low-temperature and hypoxia signals drive dynamic metabolic reprogramming via transcriptional regulation; (3) The interaction networks between key metabolites and gene modules that maintain cellular homeostasis during germination under combined stress. This research will be the first to reveal the synergistic mechanisms of DXWR’s multi-dimensional stress adaptation strategy, providing a crucial theoretical foundation and novel candidate gene resources for overcoming the bottleneck of poor germination tolerance in direct-seeded rice.

## 2. Results

### 2.1. Germination Characteristics of DXWR Under Low-Temperature and Anaerobic Conditions

**Figure 1:**
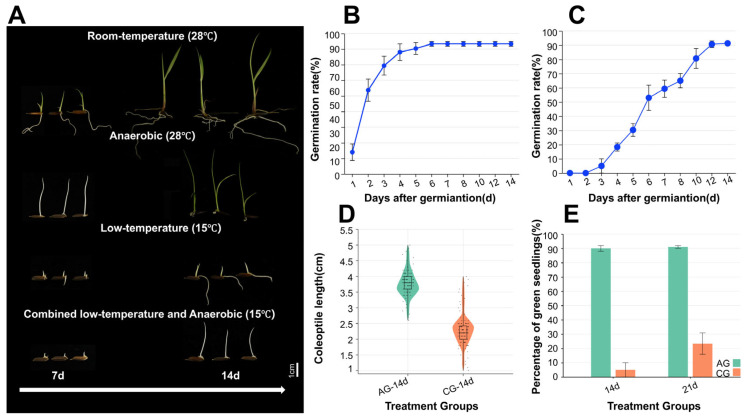
Phenotypic performance and germination metrics of DXWR under specified conditions. (**A**) Germination phenotypes at 7 and 14 days post-treatment. (**B**) Germination kinetics at 28 °C. (**C**) Germination kinetics at 15 °C. (**D**,**E**) Coleoptile length and green seedling rate under anaerobic conditions at 28 °C and combined anaerobic/low-temperature (15 °C) stress.

Under aerobic conditions, DXWR exhibited robust seed germination activity at both AG and LG conditions. At 28 °C, germination reached 80% by day 3 and was essentially complete (>95%) by day 5 (Figure 1A,B). Under the LG condition, it maintained strong germination capacity: germination initiated by day 3, reached 60% by day 7, and approached 90% by day 14 (Figure 1A,C). Remarkably, under AG conditions, germination exceeded 92% by day 14, with a green seedling rate nearing 90% and an average coleoptile length of 3.7 cm. Under CG conditions, germination exceeded 80% by day 14 with an average coleoptile length of 2.3 cm, and the green seedling rate approached 30% by day 21 (Figure 1D,E). Collectively, these findings demonstrate the exceptional low-temperature adaptation and anaerobic germination tolerance of DXWR, conferring a significant advantage for its survival and growth under abiotic stresses.

### 2.2. Gene Characteristics and Transcriptomic Responses of DXWR Under Stress

**Figure 2:**
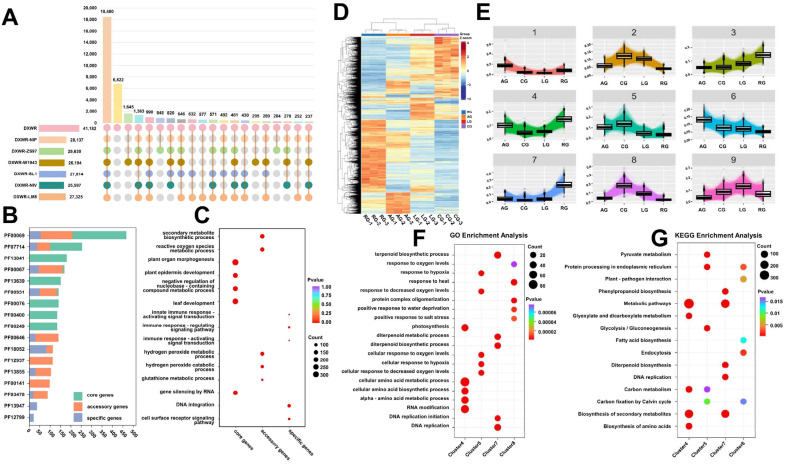
Gene characteristics, conservation, and transcriptome differences under different environments along with functional enrichment analysis of DXWR (**A**) Orthology relationships visualized through colored nodes and connecting lines (top 20 representative orthogroups shown). (**B**) PFAM family enrichment analysis of core, accessory, and specific genes in DXWR (Top10). (**C**) GO enrichment of core, accessory, and specific genes (top five most significant terms by *p*-value displayed per category). (**D**) Differentially expressed genes (DEGs) identified between stress treatments and control germination (28 °C). (**E**) Co-expression clustering of 10,593 DEGs via Coseq analysis (k = 9 clusters). (**F**,**G**) Functional enrichment analysis (Gene Ontology and KEGG pathways) for Clusters 4, 5, 7, and 8, with the top five most significant terms per category displayed.

Comparative analysis of whole-genome protein sequences between DXWR and six rice varieties categorized DXWR genes into three groups based on cross-genome conservation: core genes (18,480 orthologs shared by all seven genomes), accessory genes (15,880 with partial conservation), and unique genes (6822 specific to DXWR) (Figure 2A, Table S1). Functional enrichment revealed distinct roles: core genes, enriched in domains like Protein kinase (467) and Cytochrome P450 (169), were linked to fundamental processes (leaf development, organ morphogenesis), underscoring their role in basic growth (Figure 2B,C, Table S2). Unique genes, featuring Rx N-terminal (82) and NB-ARC (50) domains, were enriched in immune pathways, aiding pathogen defense. Accessory genes, containing Protein kinase (208) and Leucine-rich repeat (104) domains, functioned in defense and environmental adaptation, with roles in oxidative stress responses (ROS metabolism) potentially supporting anaerobic and low-temperature tolerance (Figure 2B,C, Table S2).

RNA-seq analysis of DXWR germinating embryos under low-temperature (LG), anaerobic (AG), and combined (CG) stresses revealed extensive transcriptional reprogramming. PCA showed clear separation of treatments, with PC1 (33.91%) distinguishing LG from the control (RG) and PC2 (20.32%) separating AG from other groups (Figure S4A). Using stringent thresholds (FDR ≤ 0.05, |log_2_ Fold Change| ≥ 1), 10,593 differentially expressed genes (DEGs) were identified relative to RG (Figure 2D, Table S3). CG induced the most pronounced changes (5573 upregulated, 5809 downregulated), exceeding the additive effects of individual stresses, while AG and LG elicited 2715/3560 and 3432/4244 DEGs, respectively. A comprehensive multi-omics comparison for each stress condition against the control is presented in Supplementary Figure S1 (AG vs. RG), S2 (LG vs. RG), and Figure S3 (CG vs. RG). These figures integrate transcriptomic and metabolomic data to visualize the scale of differential expression, highlight key altered molecules, and reveal coordinated pathway changes under each stress.

Coseq clustering of 10,593 DEGs yielded nine clusters with distinct expression patterns (Figure 2E, Table S3). Functional enrichment analysis of the four most stress-responsive clusters (4, 5, 7, 8) revealed distinct adaptive strategies (Figure 2F,G). Cluster 4 (synergistically repressed under combined stress) showed enrichment in anabolic processes like photosynthesis and amino acid biosynthesis, indicating a down-regulation of energy-costly functions. In contrast, Cluster 5 (specifically induced by anaerobic stress) was enriched for hypoxia response and glycolytic pathways, facilitating anaerobic energy production. Cluster 7 (co-repressed under combined stress) was linked to DNA replication and terpenoid biosynthesis, suggesting a slowdown in cell division and secondary metabolism. Conversely, Cluster 8 (synergistically induced by both stresses) was markedly enriched in broad-spectrum defense responses, including heat, salt, and plant–pathogen interaction pathways. This delineates a transcriptional network where DXWR reconfigures its physiology by repressing growth-related processes while activating specific stress-defense and energy-metabolism pathways.

### 2.3. Multi-Layered Gene Co-Regulation Mechanism of Low-Temperature and Anaerobic Composite Stress During Germination of DXWR

**Figure 3:**
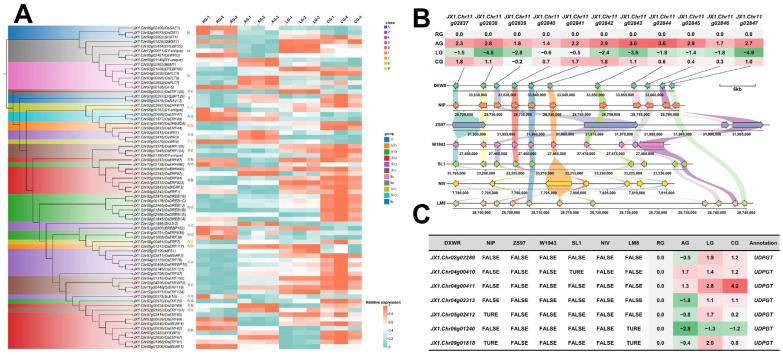
Evolution and expression of the ERF family, collinearity of the *GH18* tandem cluster, and expression patterns of *UDPGTs*. (**A**) Evolutionary relationships and expression differences in the *ERF* gene family under different conditions. (**B**) Genomic collinearity analysis of the *GH18* tandem repeat gene cluster. (**C**) Expression pattern analysis of UDP-glycosyltransferase genes (*UDPGTs*).

Analysis of the 10,593 differentially expressed genes (DEGs) identified under the control condition (RG) revealed 607 transcription factors (TFs). Among these, members of the ERF family were particularly prominent, with 72 representatives (Figure 3A), indicating a functional differentiation of genes across evolutionary hierarchies in environmental stress responses. Among core conserved genes, 69 of the 72 ERF members showed high conservation across seven rice genomes (Table S1). These genes, including members of Cluster 8 (*OsDREB1A/B/C/G*) and Cluster 2 (*OsDREB2A*), were sensitive to both LG and AG single stresses and exhibited significant synergistic induction under CG, suggesting their role as core regulatory hubs integrating diverse environmental signals to maintain germination under multi-stress conditions. Among accessory genes, the *GH18* tandem repeat gene cluster on chromosome 11 displayed unique evolutionary characteristics: highly conserved across genomes except ZS97, its expression was significantly induced by anaerobic stress but strongly repressed by low-temperature stress, indicating a potential bidirectional regulation mechanism for specific adaptation to different stresses (Figure 3B). The absence of this cluster in the ZS97 genome may correlate with its differential anaerobic tolerance during germination. At the specific gene level, seven *UDPGT* genes were identified as nearly exclusive to the DXWR genome, with expression significantly activated under single low-temperature or anaerobic stress and further enhanced under combined stress (Figure 3C). This coupling of genome specificity and stress synergistic response suggests *UDPGT* genes serve as key genetic determinants for DXWR’s unique low-temperature-anaerobic dual tolerance.

Collectively, this study systematically elucidated the molecular mechanisms of multi-level genetic elements synergistically coping with composite environmental stresses during rice germination by dissecting cross-genomic regulatory networks of core conserved genes, lineage-dependent expression patterns of partially shared genes, and cultivar-specific functions of unique genes, providing a theoretical foundation for crop stress-resistant genetic improvement.

### 2.4. Differential Accumulation and Regulatory Patterns of Stress-Responsive Metabolites

Widely targeted metabolomic profiling using UPLC-ESI-MS/MS characterized metabolic changes across the RG, AG, LG, and CG groups, with three biological replicates per group (*n* = 12 samples; Table S3). Principal Component Analysis (PCA) revealed distinct metabolic profiles among the treatment groups (Figure S4B). Principal components 1, and 2 explained 27.49%, 18.46% of the total variance, respectively. RG samples clustered tightly, indicating metabolic stability. AG samples showed clear separation from RG along PC1, highlighting significant metabolic alterations induced by anaerobic stress. LG samples exhibited dispersion within the PCA space, reflecting heterogeneous metabolic responses to low-temperature stress. CG samples partially overlapped with RG but displayed distinct separation, suggesting a unique metabolic signature under combined stress.

**Figure 4:**
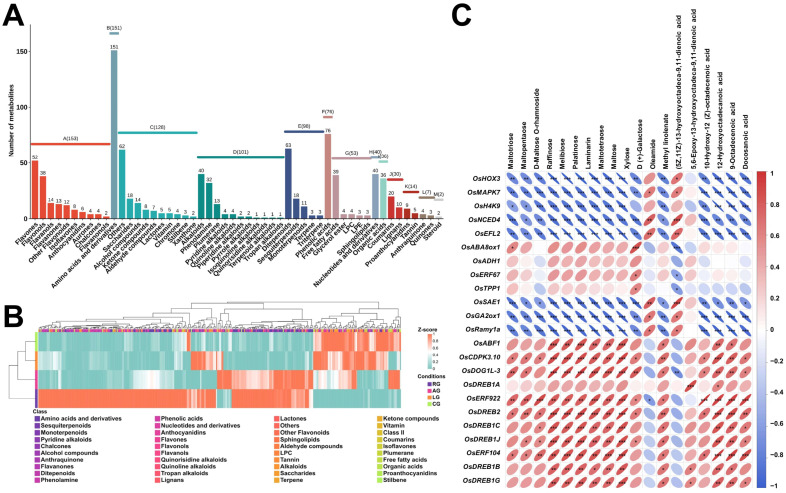
Functional classification of differentially accumulated metabolites, expression heatmap of core metabolites, and correlation analysis between seed germination-related genes and metabolites. (**A**) Hierarchical classification of differentially accumulated metabolites (DAMs) into 13 functional categories: A-M represent flavonoids, amino acids and derivatives, other minor classes, alkaloids, terpenoids, phenolic acids, lipids, nucleotides and derivatives, organic acids, lignans and coumarins, tannins, quinones, and steroids, respectively. (**B**) Expression heatmap of 348 core stress-responsive metabolites, screened from DAMs as differentially accumulated under ≥2 stress conditions and relevant to seed germination (Table S4). Color intensity (blue to red) indicates relative accumulation levels, with rows as metabolites and columns as experimental groups. (**C**) Correlation analysis between seed germination-related genes and metabolites. Pearson correlation coefficients are shown (red: positive correlation with consistent trends; blue: negative correlation with opposite trends). The ellipse shape reflects the absolute correlation value (thinner ellipses indicate higher absolute coefficients). Significance is marked as follows: * *p* < 0.05, ** *p* < 0.01, *** *p* < 0.001.

Differential Accumulated Metabolites (DAMs) were identified using a threshold of |VIP| ≥ 1, yielding 889 metabolites classified into 13 functional categories (Figure 4A). Flavonoids (153 compounds), amino acids and derivatives (151 compounds), and alkaloids (101 compounds) represented the most abundant categories, underscoring their potential significance in stress adaptation. Further screening identified 348 core stress-responsive metabolites differentially accumulated under at least two stress conditions (Figure 4B, Table S4). Within amino acid derivatives (80 compounds), 74 metabolites (92.5%) were significantly downregulated under combined stress; however, specific peptides, including Gly-Val-Ala and L-asparagine, exhibited stress-induced accumulation, suggesting specialized roles in tolerance. Among phenolic acids (35 compounds), 26 (74.3%) were universally downregulated across stress conditions.

Notably, three compounds, particularly 2-O-(4-carboxyphenethyl)-6-O-caffeoylglucoside, accumulated specifically under combined stress, indicating a potential combined-stress-specific response, while ethyl ferulate exhibited an antagonistic pattern (induced by anaerobic stress but suppressed by low temperature). For nucleotides and derivatives (26 compounds), 13 metabolites displayed reduced abundance under stress, whereas xanthine accumulated markedly under combined stress, implying its role in combined-stress adaptation. Secondary metabolites showed divergent responses: apigenin-6-C-arabinoside was significantly upregulated by low temperature, and 5 out of 17 flavonoids exhibited combinatorial-stress-specific enrichment. Low temperature also induced significant accumulation of 10 out of 11 carbohydrates and 5 out of 8 free fatty acids. Compounds such as panose, raffinose, and docosanoic acid showed synergistic accumulation under combinatorial stress.

Integrated transcriptomic-metabolomic analysis elucidated gene–metabolite regulatory modules during stress germination (Figure 4C, Table S5). A set of genes, including homeobox genes *OsHOX3*/*OsH4K9*, kinase *OsMAPK7*, hormone-related genes (*OsNCED4* [ABA biosynthesis], *OsGA2ox1* [GA catabolism]), regulatory genes (*OsEFL2*, *OsSAE1*), and the effector gene *OsRamy1a*, predominantly exhibited negative correlations with most carbohydrates and lipids. Conversely, transcription factors (*OsDREB1A/1B/1C/1G/1J/2*, *OsERF922/104*), the ABA signaling-related TF *OsABF1*, calcium-dependent kinase *OsCDPK3.10*, and seed dormancy regulator *OsDOG1L-3* showed significant positive correlations with these metabolites. Hormone pathway genes displayed distinct linkage patterns; for instance, the ABA synthesis-related gene *OsNCED4* correlated negatively, while the ABA signaling gene OsABF1 correlated positively with carbohydrates and lipids. Stress-responsive genes from diverse functional families (transcriptional regulation, signal transduction, dormancy regulation) consistently exhibited divergent correlation trends with these metabolite classes. The carbohydrate metabolites such as Raffinose, Melibiose, and Palatinose served as critical hubs across all gene regulatory categories. This modular coordination indicates that synergistic regulation by functionally diverse stress-responsive genes reconfigures metabolic networks to enhance stress adaptation during germination.

### 2.5. Coordinated Environmental Regulation of Secondary Metabolism

Integrated metabolome-transcriptome analysis (*p* < 0.05) across AG, LG, CG, and RG conditions identified 13 conserved pathways reflecting coordinated metabolic-transcriptional responses to environmental stress (Figures S1–S3). Strikingly, primary metabolism dominated these conserved pathways (92.3%, 12/13), encompassing six amino acid pathways (e.g., arginine biosynthesis; alanine, aspartate, and glutamate metabolism) and six energy–carbon pathways (e.g., pyruvate metabolism; 2-oxocarboxylic acid metabolism), highlighting the robustness of core metabolic networks. Among these, sinapic acid derivative biosynthesis showed specific co-enrichment (Rich Factor > 0.5) in AG and CG groups, suggesting its role in reinforcing cell wall structure via lignin precursor regulation during biotic stress adaptation.

Environment-specific profiling revealed divergent pathway activation patterns: AG uniquely enriched flavonoid biosynthesis pathways (luteolin aglycone, apigenin C-glycoside), whereas LG specifically activated nitrogen metabolism and fatty acid biosynthesis. Terpenoid biosynthesis (diterpenoid, ubiquinone) was co-enriched in both AG and LG, indicating potential roles in light stress adaptation.

**Figure 5:**
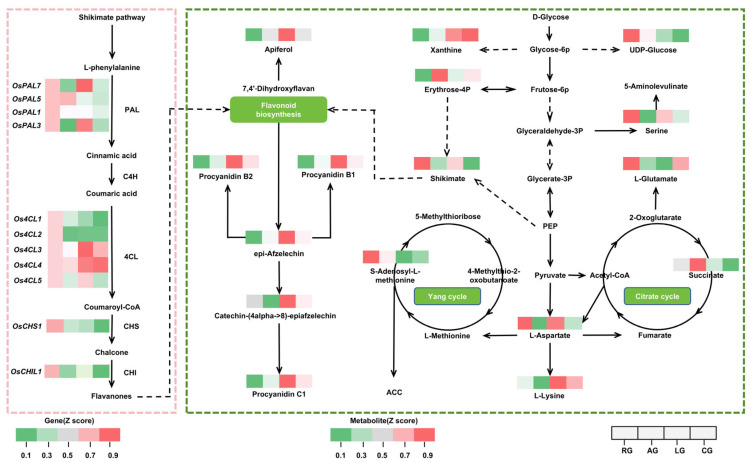
Coordinated regulation of secondary metabolism gene expression and metabolite networks. (**Left**) Expression patterns of key shikimate pathway genes under divergent conditions. (**Right**) Differential accumulation patterns of secondary metabolites across stress treatments. Dashed lines indicate indirect effects, while solid lines represent direct relationships.

Shikimate pathway regulation, a central hub for secondary metabolism, exhibited significant environmental divergence (Figure 5). *OsPAL3/7* upregulation under LG drove L-phenylalanine conversion to cinnamic acid, while OsPAL5 induction in RG/AG initiated phenylpropanoid metabolism. Differential expression of the *Os4CL* family (*Os4CL1/2* highly expressed in RG; *Os4CL3/4* induced under combined stress) collectively promoted coumaroyl-CoA generation. Concomitant upregulation of *OsCHS1* and *OsCHI1* in AG facilitated chalcone-to-flavanone conversion, aligning with flavonoid pathway enrichment (Figure 5).

Flavonoid profiling corroborated these regulatory patterns: proanthocyanidin B2 intermediate and proanthocyanidin B1 accumulated under low temperature, whereas epicatechin and proanthocyanidin C1 were enriched specifically in LG—demonstrating branch-specific suppression under anaerobic stress but induction under low-temperature conditions. Coordinated regulation through associated metabolic cycles was evident: reduced S-adenosyl-L-methionine (Yang cycle) abundance in RG/CG potentially suppressed methylation, while succinate (TCA cycle) accumulation in RG/AG suggested active energy metabolism supporting secondary biosynthesis. Xanthine accumulation and elevated UDP-glucose in RG indicated roles in secondary metabolite homeostasis and glycosylation (Figure 5).

Collectively, RG/AG conditions promoted flavonoid metabolism through key gene activation and energy provision, whereas LG conditions suppressed central flux while inducing specific product accumulation, forming an adaptive regulatory network tuned to environmental constraints.

## 3. Discussion

As the northernmost distributed wild rice species in China (28°14′ N), Dongxiang wild rice (DXWR) has been persistently exposed to combined low-temperature and anaerobic stress in its subtropical monsoon swamp habitat, leading to the evolution of a highly efficient adaptive regulatory system. Recognized as a prominent wild rice resource with exceptional tolerance to combined stress, DXWR provides an ideal model for deciphering plant adaptation mechanisms to complex environmental constraints. Through integrated genomic, transcriptomic, and metabolomic analyses, this study systematically elucidates, for the first time, the molecular mechanisms underlying DXWR’s response to combined stress during germination, revealing a multi-level adaptive framework involving synergistic “genetic regulation–metabolic response–hormonal coordination”. These findings not only fill a critical knowledge gap regarding the combined stress adaptation mechanisms in wild rice but also provide theoretical support and practical targets for breeding stress-resistant direct-seeded rice based on elite wild genetic resources.

### 3.1. A Hierarchical Genetic Framework: The Genetic Basis of DXWR’s Extreme Stress Tolerance

The exceptional tolerance of DXWR to combined stress relies on a hierarchical genetic framework, defined by comparative genomics, comprising “core genes—accessory genes—unique genes” (Figure 2A–C). This framework aligns closely with the pan-genome adaptation hypothesis, which posits that environmental adaptability in wild crop relatives stems from the synergistic interaction between conserved core pathways and lineage-specific genetic variations [36,37,38]. Our study identified 18,480 core genes, 15,880 accessory genes, and 6822 unique genes in the DXWR genome, representing distinct evolutionary categories with specialized functional roles in stress adaptation, forming a “conserved–specialized–innovative” functional division of labor.

Core genes act as central integrators of stress signals, with the highly conserved ERF transcription factor family playing a key regulatory role. As plant-specific transcriptional hubs, *ERFs* coordinate metabolic reprogramming by co-activating glycolysis, gluconeogenesis, and antioxidant pathways, facilitating both the efficient shift from oxidative phosphorylation to anaerobic respiration and the maintenance of cellular homeostasis through reactive oxygen species (ROS) scavenging [39,40,41,42,43,44]. Among the 72 identified ERF members, 69 were conserved across the seven rice genomes (Table S1), including core members of the DREB subfamily (e.g., OsDREB1A/B/C/G in Cluster 8 and *OsDREB2A* in Cluster 2). These genes exhibited sensitivity to both single stresses, LG and AG, and demonstrated synergistic induction under CG (Figure 3A). The observed synergistic induction pattern of these *ERF/DREB* family genes under combined stress (Figure 3A) suggests their enhanced capability to coordinately activate downstream stress resistance pathways, forming a more efficient regulatory mode for responding to complex stress conditions [45,46,47]. This characteristic is highly adapted to the long-term selective pressures of its native habitat and represents a key manifestation of its progressively strengthened stress adaptability.

Accessory genes confer specialized adaptive functions to DXWR, typified by the *GH18* glycoside hydrolase tandem gene cluster on chromosome 11. This cluster is conserved in wild rice and temperate japonica varieties but is absent in the anaerobic-sensitive indica cultivar ZS97. It exhibits a bidirectional regulatory pattern of “anaerobic induction—low temperature suppression”, being significantly upregulated under AG and downregulated under LG (Figure 3B). The combination of this expression pattern and functional conservation suggests that the *GH18* cluster dynamically optimizes energy allocation according to stress type—promoting coleoptile elongation under AG (supporting DXWR’s 3.7 cm coleoptile length, Figure 1D) to escape hypoxia via cell wall chitin hydrolysis and signal transduction, while suppressing its expression under LG to conserve energy for core metabolic processes, perfectly matching survival needs under different stresses [48,49].

Unique genes further highlight DXWR’s adaptive specificity. Seven DXWR-specific *UDPGT* genes showed significant stress-responsive characteristics: activated expression under single stresses and synergistically upregulated under CG (Figure 3C). For instance, *JX1.Chr04g00411* exhibited a log_2_FC of 4.2 under CG, far exceeding its levels under single stresses. These *UDPGT* genes may enhance stress resistance by remodeling glycosylation patterns and cell wall structure [13,49,50,51,52]. Metabolomic data corroborated this, showing a concurrent 1.9-fold increase in jasmonic acid-glucoside under CG in DXWR (Table S4), supporting their role in regulating hormone homeostasis through hormone glycosylation. This mechanism directly contributes to DXWR’s 30% green seedling rate under CG (Figure 1E).

### 3.2. Metabolic Reprogramming: Synergistic Energy Optimization and Antioxidant Defense

**Figure 6:**
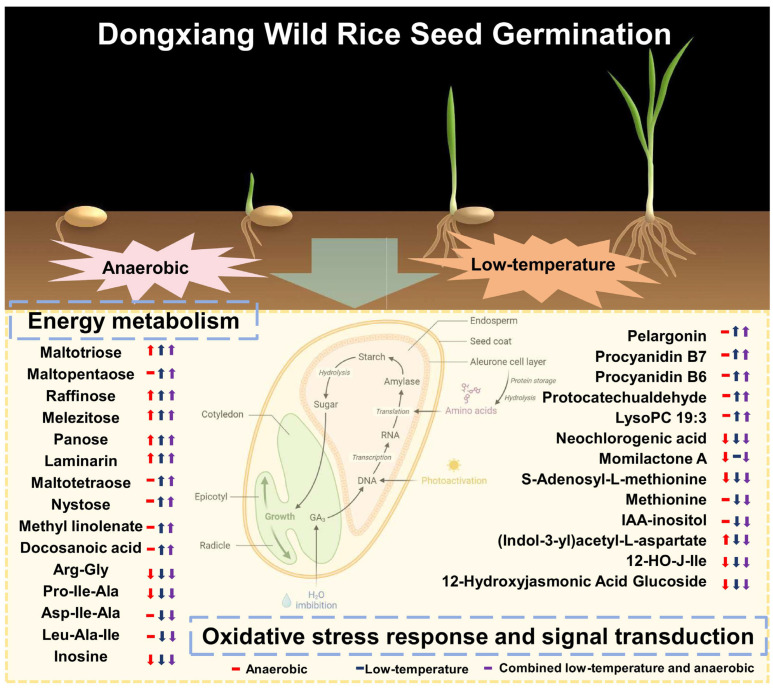
Multidimensional adaptation system of “Energy optimization—Redox balance—Growth inhibition mitigation” for low-temperature anaerobic germination in DXWR.

Integrated metabolomic and transcriptomic analyses reveal that DXWR employs precise metabolic reprogramming to construct a physiological adaptation strategy synergistic between “energy assurance” and “antioxidant defense” (see the “Metabolic Reprogramming Synergistic Module” in Figure 6), a process central to its chemical adaptation to combined stress.

Energy optimization centers on a “multi-carbon reserve + alternative metabolism” strategy. Under CG, DXWR accumulates not only raffinose, panose, and maltotriose but also other oligosaccharides like maltopentaose and stachyose (Table S4). These oligosaccharides serve as stable carbon reservoirs and can be rapidly broken down into glucose, providing substrates for the glycolytic pathway under hypoxia—the core alternative pathway for ATP synthesis when oxidative phosphorylation is impaired [53,54,55]. Transcriptomic data further confirm the significant enrichment of genes related to oligosaccharide synthesis, glycolysis/gluconeogenesis under CG (Figure 2G, Table S3), forming a linked mechanism of “metabolite accumulation—pathway activation”. Concurrently, the catabolism of tripeptides (e.g., Pro-Ile-Ala, Asp-Ile-Ala, Leu-Ala-Ile) under CG (Table S4) releases free amino acids like leucine and isoleucine, replenishing tricarboxylic acid cycle intermediates and further enhancing energy production under nutrient-limited conditions [56,57].

Antioxidant defense relies on the targeted construction of a “ROS scavenging network + signal regulation”. Under CG, DXWR accumulates not only procyanidin B7, pelargonidin, and neochlorogenic acid but also substances like procyanidin B6, protocatechualdehyde, and lyso-phosphatidylcholine (LysoPC 19:3) (Table S4). These compounds are efficient ROS scavengers, reducing oxidative damage by providing hydrogen atoms or inhibiting ROS-generating enzyme activity, and may also participate in germination signaling regulation [54,55]. Simultaneously, key genes in the phenylpropanoid pathway show stress-specific upregulation: *OsPAL3/7* are activated under LG; *Os4CL3/4* are upregulated under CG; and *OsCHS1/OsCHI1* are activated under AG/CG (Figure 5), achieving precise matching of “stress type—gene expression—metabolite accumulation” [58]. Notably, the accumulation of antioxidant metabolites under CG significantly exceeds that under single stresses (only 1.2–1.5-fold increases under LG/AG), indicating that DXWR can perceive the intensity of combined stress and reinforce its antioxidant defense through metabolic reprogramming. Furthermore, efficient ROS scavenging protects glycolytic enzymes from oxidative inactivation, creating a positive feedback loop between energy metabolism and redox balance, providing dual protection for survival under combined stress.

### 3.3. Gene–Metabolite Networks and Hormonal Crosstalk: Fine-Tuning the Growth–Defense Balance

Correlation analysis reveals that DXWR constructs a dynamic balance system prioritizing “stress adaptation over basic growth maintenance” through modular coordination within gene–metabolite networks (see the “Gene–Metabolite–Hormone Regulatory Network Module” in Figure 6). Hormonal crosstalk further refines this balance through precise regulation.

In the ABA pathway, under combined stress (CG), the key ABA biosynthesis gene *OsNCED4* is significantly downregulated (log_2_FC = −1.2, Supplementary Table S3), potentially reducing excessive ABA accumulation that leads to germination arrest [59]. Concurrently, the core ABA signaling transcription factor *OsABF1* is upregulated (log_2_FC = 1.5, Supplementary Table S3), ensuring the retention of ABA-responsive functions related to low-temperature defense [60]. This results in a regulatory outcome where “stress resistance capacity is maintained without impeding the germination process”. In the JA pathway, the content of 12-hydroxyjasmonic acid glucoside increases 2.1-fold under CG compared to the control (Supplementary Table S4), and the seven DXWR-specific UDPGT genes (e.g., JX1.Chr04g00411) show synergistic upregulation (log_2_FC = 4.2, Supplementary Table S3). Considering the known role of the *UDPGT* family in mediating hormone glycosylation, it is hypothesized that this process converts active JA into a stored glycoside form. This could potentially allow for the release of active JA via hydrolysis to enhance defense signals later, thereby avoiding excessive JA-induced growth inhibition while reserving signaling molecules for persistent stress. This regulation provides hormonal-level support for DXWR’s maintained 2.3 cm coleoptile elongation under CG (Figure 1D) [61,62]. In the auxin pathway, the content of auxin conjugates like IAA-aspartate increases 1.7-fold under CG (Supplementary Table S4). Such conjugates can reduce free auxin activity, potentially delaying radicle emergence to conserve energy, aligning with the “survival-first” adaptation strategy [63].

Furthermore, the reduced content of S-adenosyl-L-methionine (SAM) under CG (Supplementary Table S4), a key methyl donor for DNA methylation, might induce genomic DNA hypomethylation, potentially releasing the repression of stress-responsive genes [64]. This change is associated with the enrichment of the endoplasmic reticulum protein processing pathway, providing epigenetic-level synergy for transcriptional regulation. Ultimately, this forms a multi-level adaptive network linking “genetic regulation—epigenetic modification—metabolic response”, further strengthening DXWR’s tolerance to combined stress.

### 3.4. Implications for Breeding Climate-Adapted Direct-Seeded Rice

The hierarchical genetic framework and metabolic strategies of DXWR provide potential directions for exploring breeding approaches to address the combined low-temperature and anaerobic stress faced by direct-seeded rice in early spring. These can be preliminarily summarized into two potential research paths: First, exploring the introduction of the *GH18* tandem gene cluster (conserved in wild rice but absent in cultivated rice) into anaerobic-sensitive varieties, aiming to mimic DXWR’s phenotype of 3.7 cm coleoptile elongation under AG conditions (Figure 1D). The potential mechanism may involve the optimization of cell wall modification and energy allocation, potentially partially enhancing seedling escape capacity under flooding, thereby alleviating the “seedling submergence death” problem. Second, exploring the functional modification using DXWR-specific *UDPGT* genes like *JX1.Chr04g00411*, leveraging their potential involvement in hormone glycosylation pathways to regulate hormone homeostasis, which might help improve the green seedling rate under CG conditions. Additionally, the synergistic induction characteristics exhibited by genes like *OsDREB1A/B* under CG (Table S3) could be further evaluated in the future for their potential as auxiliary screening markers for stress-tolerant lines. These strategies hold promise for complementing traditional quantitative trait locus (QTL) mapping methods at the functional gene level, providing theoretical reference and genetic resources for future breeding of “climate-smart” rice varieties adapted to complex stresses like low temperature and waterlogging in the context of climate change, possessing certain exploratory value.

## 4. Materials and Methods

### 4.1. Plant Materials and Growth Conditions

DXWR seeds were obtained from the core DXWR germplasm population maintained by the Jiangxi Academy of Agricultural Sciences, China (28°14′ N). To minimize genetic variability, seeds used in this study were derived from a single DXWR line that had been stabilized through multiple generations of bagging self-pollination. Mature seeds were harvested 45 days after heading, air-dried at ambient temperature, and subsequently treated at 50 °C for 7 days to break dormancy before experimentation. Three independent biological replicates (50 seeds per replicate) were surface-sterilized with 1.5% (*v*/*v*) sodium hypochlorite for 30 min, followed by three rinses with sterile distilled water to remove residual disinfectant, and air-dried prior to experimentation [21].

### 4.2. Germination Treatments and Experimental Design

Seeds were subjected to four germination treatments under a 12/12 h photoperiod (light intensity: 3000 lux): Room-temperature aerobic germination (RG): Seeds were placed in 10 cm diameter Petri dishes containing 10 mL sterile water and incubated at 28 °C ± 1 °C. Low-temperature aerobic germination (LG): As above, but incubated at 15 °C ± 1 °C to simulate cold stress. Room-temperature anaerobic germination (AG): Seeds were completely submerged in sterile water within airtight glass bottles (4 cm diameter × 20 cm height) and incubated at 28 °C. Combined low-temperature and anaerobic germination (CG): Seeds were submerged in sterile water in airtight bottles and incubated at 15 °C to impose dual stress [1,21].

### 4.3. Germination Kinetics and Phenotypic Analysis

Germination assays were performed in triplicate (n = 3). Seed germination was monitored daily for 14 days, with germination defined as coleoptile elongation exceeding 2 mm. Germination rate (GR) was calculated as: GR (%) = Number of germinated seeds/Total seeds per replicate 100%. Data were recorded at 24 h intervals to construct germination curves [1].

### 4.4. Sample Collection for Multi-Omics Analysis

Based on prior studies indicating peak transcriptomic and metabolomic activity at 12 h post-imbibition [65], embryos were dissected from seeds treated under RG, LG, AG, and CG conditions at this time point. To minimize endosperm contamination, embryos were rapidly excised under a stereomicroscope, flash-frozen in liquid nitrogen, and stored at −80 °C. Each treatment included three biological replicates (1000 mg fresh weight per sample), prepared for subsequent RNA sequencing (RNA-seq) and liquid chromatography–mass spectrometry (LC-MS)-based metabolomics analysis.

### 4.5. Orthologous Gene Identification

This study integrated whole-genome protein sequences from seven representative rice accessions: Dongxiang wild rice (*Oryza rufipogon*; DXWR), cultivated japonica rice (Oryza sativa ssp. japonica cv. Nipponbare), indica rice (*O. sativa* ssp. *indica* cv. ZS97), common wild rice (*O. rufipogon*; W1943), Sri Lankan common wild rice (*O. rufipogon*; SL1), *Oryza nivara*, and the broad-spectrum resistance germplasm LM8. Genomic datasets were obtained from public repositories: Ensembl Plants (https://plants.ensembl.org/ (accessed on 10 June 2025)) and the Rice Germplasm Genome Database (http://www.ricegermplasmgenome.com/ (accessed on 27 June 2025)). All-vs-all protein sequence alignment was performed using OrthoFinder (v2.5. 5) to generate a homology network based on BLASTP e-values. Gene family clusters (Orthogroups) were delineated through the Markov Clustering algorithm (MCL; inflation = 1.5). To accurately distinguish orthologs from paralogs, a species tree was constructed from single-copy orthologous genes using the STAG algorithm, followed by STRIDE algorithm-mediated integration of gene tree-species tree concordance analysis to refine homology assignments. Orthologs were defined as homologous genes originating from speciation events, while paralogs were defined as those arising from gene duplication events [66].

### 4.6. RNA-Seq Analysis: Sampling, Library Preparation, Sequencing, and Bioinformatics

Total RNA was extracted from seed embryos of the collected samples and subjected to rigorous quality control. Qualified RNA samples were used to construct cDNA libraries, which were subsequently sequenced on an Illumina platform employing high-throughput sequencing technology. Raw sequencing reads underwent quality control, filtering, and de novo transcriptome assembly to generate transcript sequences. Processed reads were aligned to the reference genome, and gene expression levels were quantified. Differentially expressed genes (DEGs) between anaerobic/low-temperature treatment groups and the control group were identified using DESeq2 with stringent thresholds (|log_2_ Fold Change| > 1 and adjusted *p*-value < 0.05). To analyze co-expression networks of germination-related genes in DXWR, expression data were normalized using arcsine transformation and analyzed within the DIANE framework [67]. A multifactorial model was applied, and modules were partitioned using the Conseq algorithm with an optimal cluster number (k = 9). DEGs were functionally annotated against the Gene Ontology (GO) and Kyoto Encyclopedia of Genes and Genomes (KEGG) databases, and enrichment analysis was performed to elucidate their primary biological functions and associated metabolic pathways. Transcriptomic data analysis, visualization (including heatmaps and functional enrichment plots), and genomic collinearity analysis were performed using the online platforms Chiplot (https://www.chiplot.online (accessed on 5 August 2025)) and MetWare Cloud (https://cloud.metware.cn (accessed on 6 August 2025)).

### 4.7. Metabolomic Profiling and Analysis

Metabolites in seed samples were profiled using gas chromatography–mass spectrometry (GC-MS) or liquid chromatography–mass spectrometry (LC-MS). Following sample pretreatment and metabolite extraction, chromatographic separation and mass spectrometric detection yielded raw chromatographic peaks and spectral data. Raw data were processed through peak detection, alignment, and area quantification using specialized software (e.g., XCMS Online, homepage: https://xcmsonline.scripps.edu/, (accessed on 15 April 2025); MS-DIAL, homepage: https://prime.psc.riken.jp/Metabolomics_Software/MS-DIAL/, (accessed on 15 April 2025); Compound Discoverer, homepage: https://www.thermofisher.com/us/en/home/industrial/mass-spectrometry/liquid-chromatography-mass-spectrometry-lc-ms/lc-ms-software/compound-discoverer-software.html, (accessed on 15 April 2025)) to construct a metabolite dataset. Differential metabolites between anaerobic/low-temperature treatment groups and controls were identified through multivariate statistical analysis, including principal component analysis (PCA) for dimensionality reduction and partial least squares-discriminant analysis (PLS-DA) for group discrimination, applying thresholds of VIP > 1 (Variable Importance in Projection) and *p*-value < 0.05 (Student’s *t*-test). Significant metabolites were annotated by matching against standard reference databases (e.g., NIST, HMDB) or via MS/MS spectral interpretation, with subsequent metabolic pathway enrichment analysis performed using the Kyoto Encyclopedia of Genes and Genomes (KEGG) database to elucidate biological context.

## 5. Conclusions

This study systematically decrypts the multi-tiered regulatory framework conferring Dongxiang wild rice (DXWR) with remarkable resilience to combined low-temperature and anaerobic stress. By integrating multi-omics analyses, we demonstrate that this resilience stems from the synergistic interaction of a hierarchically structured genome (featuring core, accessory, and unique genes), transcriptional hubs dominated by the *ERF/DREB* subfamily, and extensive metabolic reprogramming that prioritizes energy optimization and redox homeostasis. These findings not only elucidate the molecular basis of ecological adaptation but also yield two actionable breeding strategies: utilizing the *GH18* gene cluster to enhance anaerobic energy mobilization and deploying specific *UDPGT* genes to fortify hypoxia resilience. Looking forward, this work provides a springboard for future research. The functional validation of candidate genes via gene editing, the investigation of spatiotemporal dynamics throughout germination, and the assessment of their agronomic value in field trials represent critical next steps. The genomic and metabolic resources identified here establish a foundational roadmap for developing climate-resilient, stress-tolerant rice varieties, contributing significantly to the goal of sustainable agriculture.

## Data Availability

The original contributions presented in this study are included in the article/Supplementary Material. Further inquiries can be directed to the corresponding author.

## Supplementary Materials

The following supporting information can be downloaded at: https://www.mdpi.com/article/10.3390/plants14203120/s1. Figure S1: Transcriptomic and metabolomic differences between AG and RG; Figure S2: Transcriptomic and metabolomic differences between LG and RG; Figure S3: Transcriptomic and metabolomic differences between CG and RG. Figure S4: Principal component analysis of transcriptomic and metabolomics data across groups; Table S1: Identification of orthologous genes of Dongxiang wild rice (DXWR) across seven genomes; Table S2: PFAM and GO enrichment analyses of core genes, accessory genes, and specific genes in DXWR (Top 10 enriched PFAM families; Top 5 enriched GO terms); Table S3: Expression data of 10,593 genes with differential ex-pression compared to RG, and top 5 enriched GO terms and KEGG pathways for clusters 4, 5, 7, and 8; Table S4: Comprehensive accumulation profiles and functional annotations of 348 core stress-responsive metabolites in DXWR during germination under differential stress conditions; Table S5: Pairwise Pearson correlation analysis between key germination-related genes and stress-responsive metabolites.

## Abbreviations

The following abbreviations are used in this manuscript:

DXWR	Dongxiang Wild Rice
RG	Room-temperature aerobic germination
AG	Low-temperature aerobic germination
CG	Combined low-temperature and anaerobic germination
UDPGT	UDP-Glycosyltransferase
GH18	Glycoside hydrolases 18
