# Supplementary material for: Integrated Multi-Omics Analysis Reveals the Survival Strategy of Dongxiang Wild Rice (DXWR, Oryza rufipogon Griff.) Under Low-Temperature and Anaerobic Stress
Source: Plants (Basel). 2025 Oct 10;14(20):3120. doi: 10.3390/plants14203120 (PMC12567391; doi:10.3390/plants14203120)
Supplement: Supplementary file 1 [file plants-14-03120-s001.zip › plants-3888558-Supplementary.pdf]

Supplementary Material

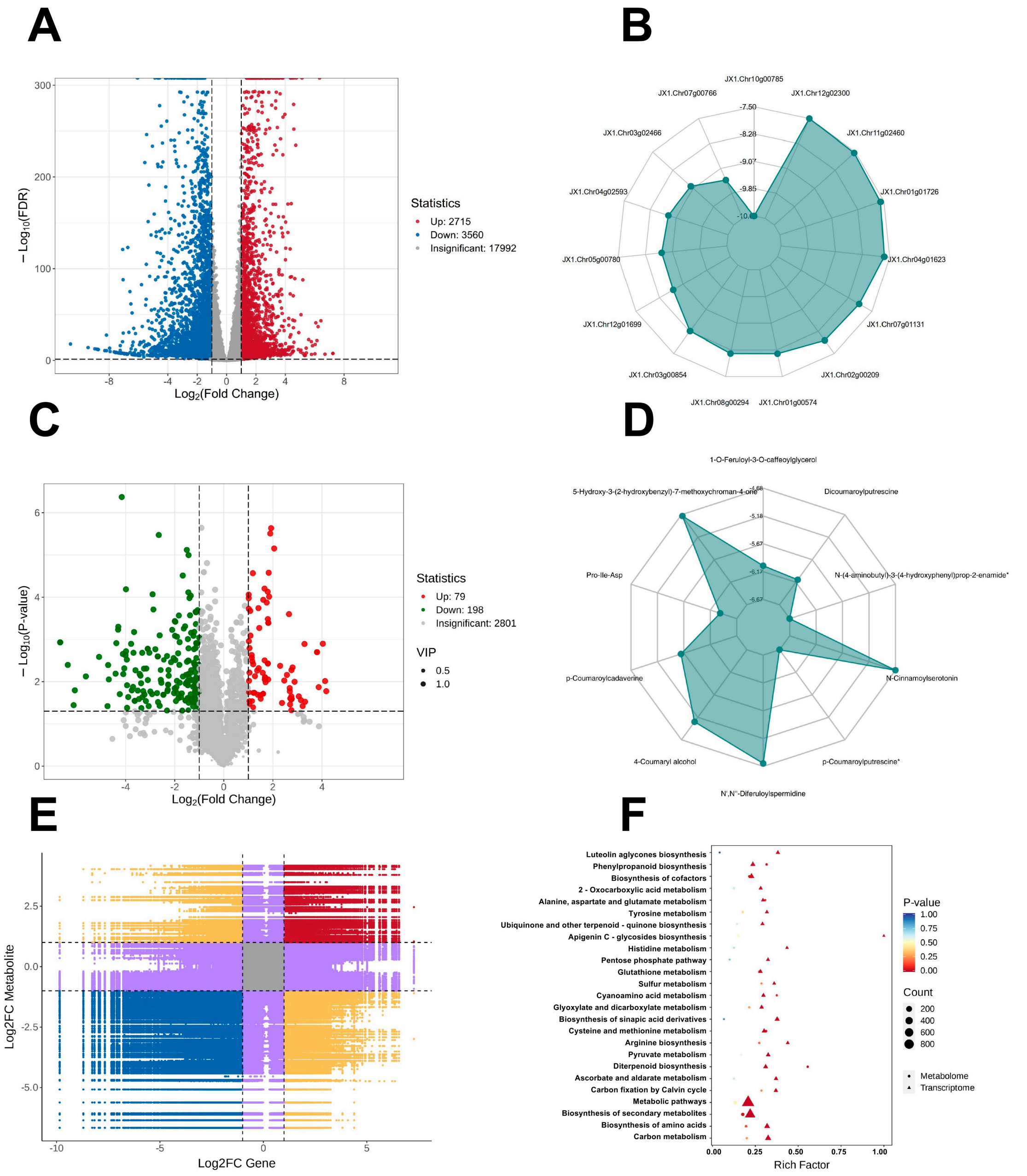

Figure S1. Transcriptomic and metabolomic differences between AG and RG.

Definitions of plot types: (A, C) Volcano plots: Display differential ex-pression/accumulation of genes (A) or metabolites (C), with $\log_2$(fold change) on the x-axis and -log10(adjusted p-value) on the y-axis; red dots indicate significantly differential items ($|\log2FC| \geq 1$, adjusted $p < 0.05$). (B and D) Radar plots: Show the top 15 differential genes (B) or top 10 differen-tial metabolites (D) ranked by |log2FC|. (E) 9-Quadrant plots: Illustrate associations between transcriptomic and metabolomic changes, with each quadrant representing a combination of up/down-regulation in genes and metabolites. (F) Integrated KEGG enrichment: Highlight significantly enriched pathways (adjusted $p < 0.05$) by overlapping differential genes and metabolites. AG: anaerobic germination at room temperature, LG: aerobic germination at low temperature.

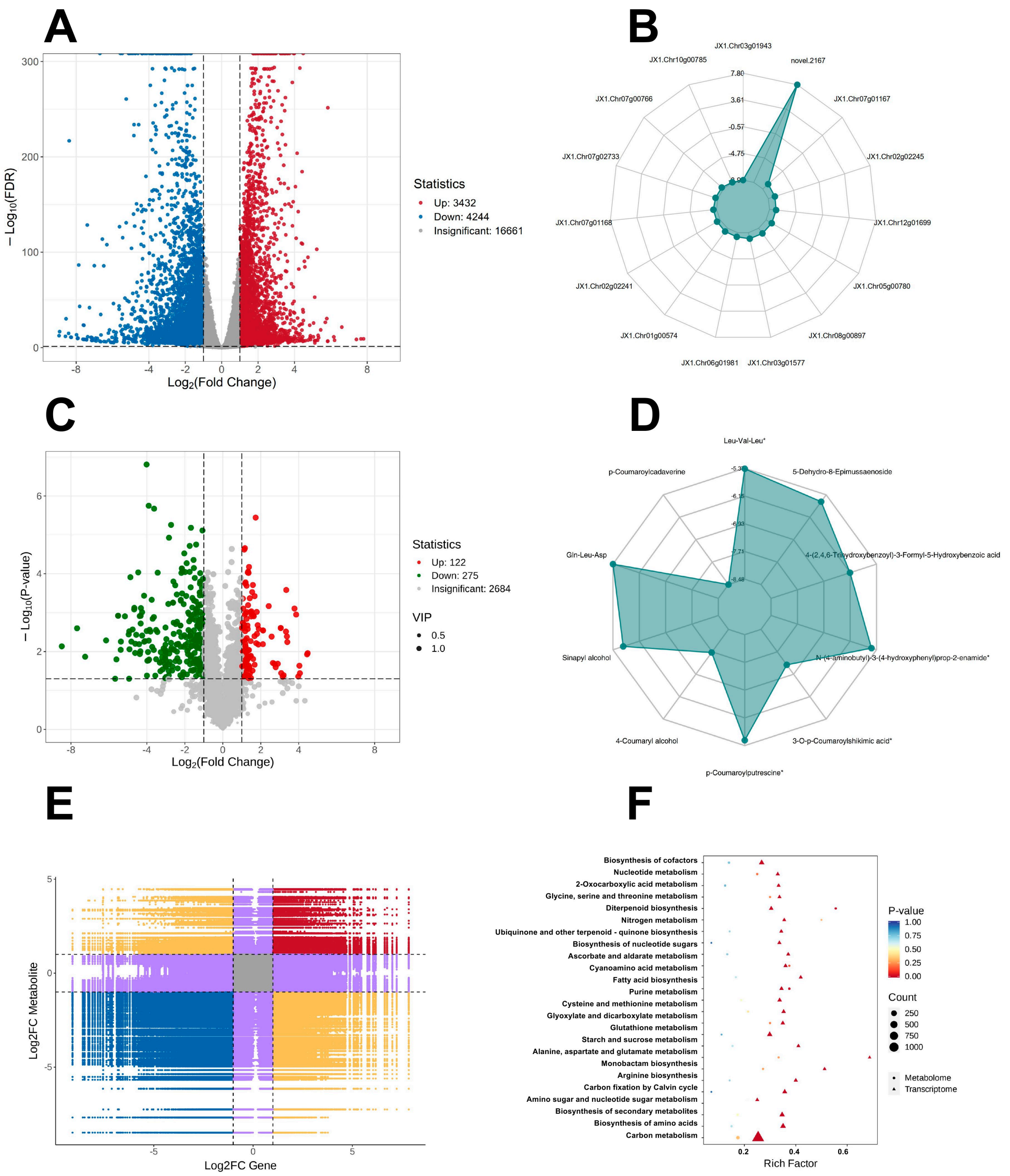

Figure S2. Transcriptomic and metabolomic differences between LG and RG.

Definitions of plot types: (A, C) Volcano plots: Display differential ex-pression/accumulation of genes (A) or metabolites (C), with $\log_2$(fold change) on the x-axis and -log10(adjusted p-value) on the y-axis; red dots indicate significantly differential items (|log2FC| ≥ 1, adjusted $p < 0.05$). (B and D) Radar plots: Show the top 15 differential genes (B) or top 10 differen-tial metabolites (D) ranked by |log2FC|. (E) 9-Quadrant plots: Illustrate associations between transcriptomic and metabolomic changes, with each quadrant representing a combination of up/down-regulation in genes and metabolites. (F) Integrated KEGG enrichment: Highlight significantly enriched pathways (adjusted $p < 0.05$) by overlapping differential genes and metabolites. LG: aerobic germination at low temperature, LG: aerobic germination at low temperature.

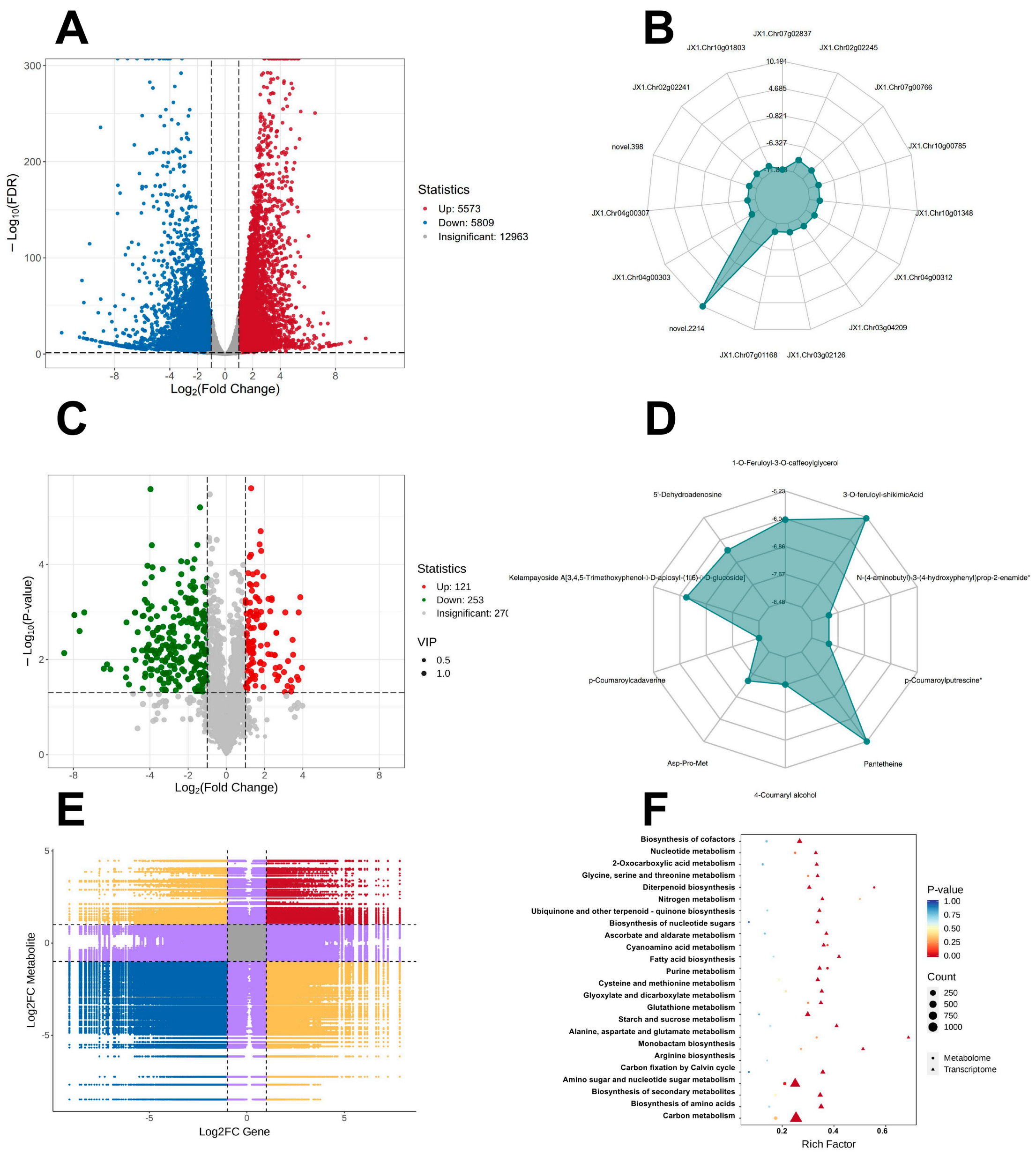

Figure S3. Transcriptomic and metabolomic differences between CG and RG.

Definitions of plot types: (A, C) Volcano plots: Display differential ex-pression/accumulation of genes (A) or metabolites (C), with $\log_2$(fold change) on the x-axis and -log10(adjusted p-value) on the y-axis; red dots indicate significantly differential items ($|\log2FC| \geq 1$, adjusted $p < 0.05$). (B and D) Radar plots: Show the top 15 differential genes (B) or top 10 differen-tial metabolites (D) ranked by |log2FC|. (E) 9-Quadrant plots: Illustrate associations between transcriptomic and metabolomic changes, with each quadrant representing a combination of up/down-regulation in genes and metabolites. (F) Integrated KEGG enrichment: Highlight significantly enriched pathways (adjusted $p < 0.05$) by overlapping differential genes and metabolites. CG: anaerobic germination at low temperature, LG: aerobic germination at low temperature.

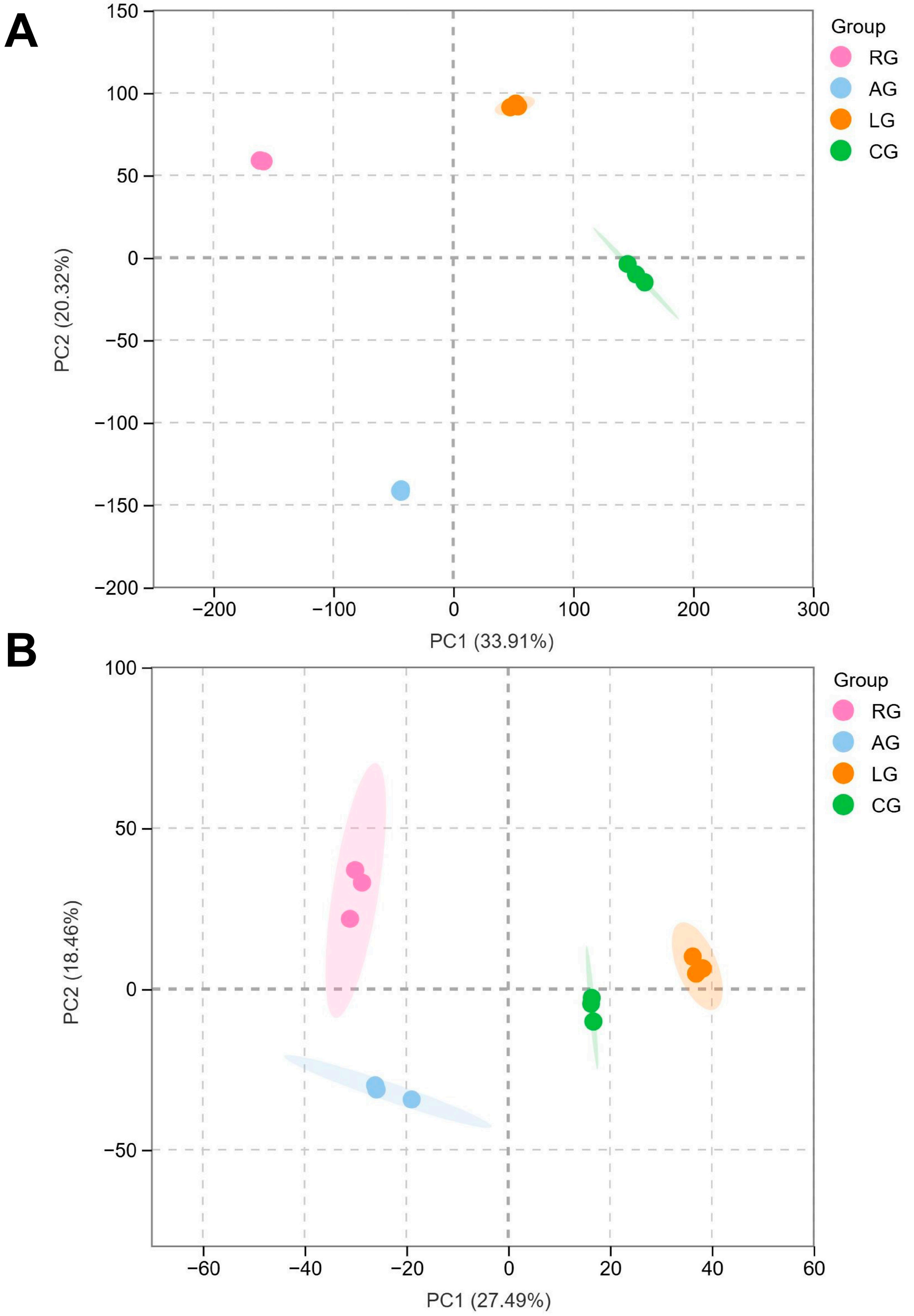

Figure S4. Principal component analysis of transcriptomic (A) and metabolomics (B) data across groups.
